# An ancillary care policy in a vaccine trial conducted in a resource-constrained setting: evaluation and policy recommendations

**DOI:** 10.1136/bmjgh-2024-015259

**Published:** 2024-06-10

**Authors:** Gwen Lemey, Ynke Larivière, Bernard Isekah Osang'ir, Trésor Zola, Primo Kimbulu, Solange Milolo, Engbu Danoff, Yves Tchuma, Vivi Maketa, Patrick Mitashi, Raffaella Ravinetto, Pierre Van Damme, Jean-Pierre Van geertruyden, Hypolite Muhindo-Mavoko

**Affiliations:** 1 Global Health Institute, University of Antwerp, Antwerpen, Belgium; 2 Centre for the Evaluation of Vaccination, University of Antwerp, Antwerpen, Belgium; 3 Tropical Medicine Department, University of Kinshasa, Kinshasa, Congo (the Democratic Republic of the); 4 Public Health, Institute of Tropical Medicine, Antwerp, Belgium; 5 School of Public Health, University of the Western Cape, Cape Town, South Africa

**Keywords:** Global Health, Vaccines, Cohort study, Health policy

## Abstract

**Introduction:**

Clear guidelines to implement ancillary care (AC) in clinical trials conducted in resource-constrained settings are lacking. Here, we evaluate an AC policy developed for a vaccine trial in the Democratic Republic of the Congo and formulate policy recommendations.

**Methods:**

To evaluate the AC policy, we performed a longitudinal cohort study, nested in an open-label, single-centre, randomised Ebola vaccine trial conducted among healthcare personnel. Participants’ demographic information, residence distance to the study site and details on the financial and/or medical support provided for any (serious) adverse events ((S)AE) were combined and analysed. To assess the feasibility of the AC policy, an expenditure analysis of the costs related to AC support outcomes was performed.

**Results:**

Enrolment in this evaluation study started on 29 November 2021. The study lasted 11 months and included 655 participants from the Ebola vaccine trial. In total, 393 participants used the AC policy, mostly for AE management (703 AE and 94 SAE) via medication provided by the study pharmacy (75.3%). Men had a 35.2% (95% CI 4.0% to 56.6%) lower likelihood of reporting AE compared with women. Likewise, this was 32.3% lower (95% CI 5.8% to 51.4%) for facility-based compared with community-based healthcare providers. The daily AE reporting was 78.8% lower during the passive vs the active trial stage, and 97.4% lower during unscheduled vs scheduled visits (p<0.001). Participants living further than 10 km from the trial site more frequently reported the travel distance as a reason for not using the policy (p<0.04). In practice, only 1.1% of the operational trial budget was used for AC policy support.

**Conclusion:**

The trial design, study population and local health system impacted the use of the AC policy. Nonetheless, the AC policy implementation in this remote and resource-constrained setting was feasible, had negligible budgetary implications and contributed to participants’ healthcare options and well-being.

**WHAT IS ALREADY KNOWN ON THIS TOPIC**

- Implementing ancillary care (AC) in clinical trials is encouraged through international guidelines but remains non-compulsory. Whereas several research groups have provided models and theoretical approaches, there is a dearth of strategies for practical implementation, thus AC is mostly defined on a case-by-case or ad hoc approach. We previously developed and published an AC algorithm and policy that was systematically applied in an Ebola vaccine trial in a remote and resource-constrained area of the Democratic Republic of the Congo.

**WHAT THIS STUDY ADDS**

- This is, to the best of our knowledge, the first study that evaluates the outcomes of a study-specific AC policy. It demonstrates the feasibility of a well-structured AC approach to provide medical and/or financial support to trial participants who experience medical events, with limited budgetary implications for the sponsor, investigators and funders. Based on the study results, that is, the participant utilisation of the AC policy and the related support outcomes, we provide recommendations to other research groups and sponsors willing to implement a similar approach.

**HOW THIS STUDY MIGHT AFFECT RESEARCH, PRACTICE OR POLICY**

- This AC approach can be adapted to other contexts and clinical trials, and inspire policy-makers, ethics committees and funders to call for adequate AC provisions in clinical trial settings with access to care constraints.

## Introduction

The development of vaccines and medicines for diseases affecting low-income and middle-income countries remains crucial to reduce global health inequalities. Ebola vaccines are primarily needed in remote areas of West and Central Africa, where outbreaks are more prevalent. Therefore, the University of Antwerp (UAntwerp; Belgium), as sponsor, and the University of Kinshasa (UNIKIN; Democratic Republic of Congo (DRC)), as principal investigator (PI), conducted a large Ebola vaccine trial in Boende, DRC, between December 2019 and October 2022. In 2014, the Boende health zone experienced an Ebola outbreak, with healthcare personnel disproportionately affected.^1^ To enhance the outbreak preparedness of this Ebola-endemic region, the trial assessed the safety and immunogenicity of a heterologous Ebola vaccine regimen (Ad26.ZEBOV, MVA-BN-Filo) in this at-risk population, with a booster dose (Ad26.ZEBOV), administered one (Arm 1) or two (Arm 2) years after the first dose (randomisation 1:1).^2^

In the early stages of the trial, the sponsor and PI were confronted with the substandard quality healthcare routinely available and accessible to the population in Boende, including the trial participants.^3^ This context-related vulnerability may impact individuals’ motivation to participate in research.^4^ However, it is important not to overemphasise the effect of structural coercion on the decision of prospective participants to enrol in clinical trials, as it underestimates the role of the individual agency. Enrolment may offer the best possible outcome for socially vulnerable participants and be an active choice, in expectation of better care.^5^ As such, researchers should take adequate measures to mitigate the effects of socioeconomic vulnerability, by promoting quality healthcare and addressing participants’ therapeutic expectations based on their specific (health) needs.^6 7^ According to the Council for International Organisations of Medical Sciences, offering medical care that goes beyond the scope of a scientific study—also referred to as ancillary care (AC)—should not be considered an undue influence to participate in research. On the contrary, it may contribute to optimising the balance between burdens and benefits of research participation.^8^ Hence, the UAntwerp and UNIKIN team developed an AC policy, to provide medical and/or financial support for all medical events experienced by trial participants, irrespective of their relatedness to the investigational product (IP) or to trial procedures, which has been published elsewhere.^3^

In this paper, we share the findings from the assessment of this trial-specific AC policy, which aimed to treat and financially cover the treatment expenses of participants’ unrelated ((serious) adverse event (S)AE). The primary objective was to evaluate the participants’ use of the AC policy, including its policy outcomes in terms of medical and/or financial support, geographic determinants, budgetary implications and formulate recommendations for AC strategies in similar settings.

## Methods

### Study design and participants

**Figure 1:**
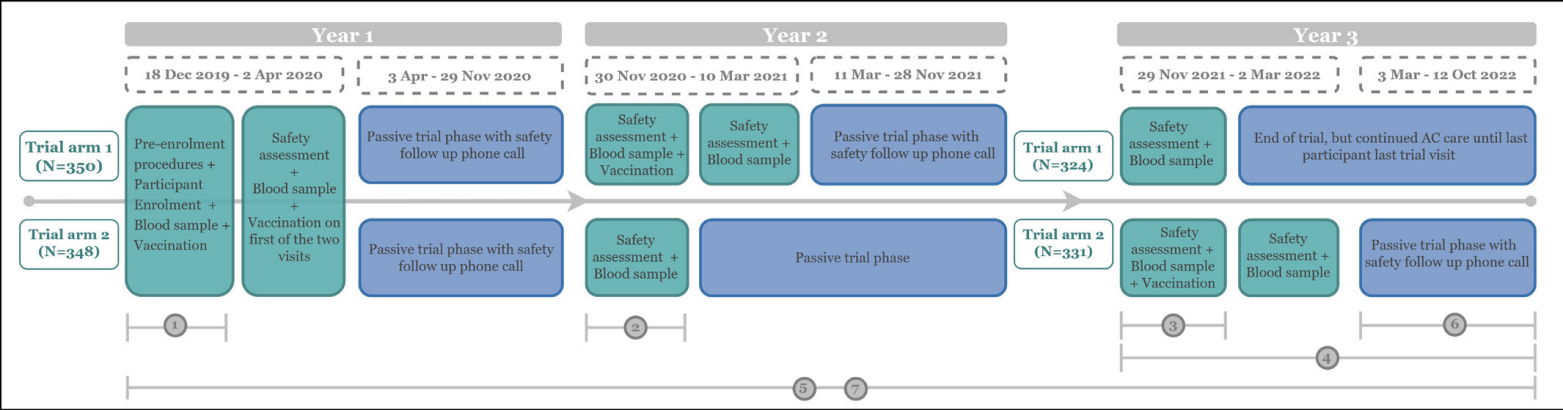
Overview of the study design of the Ebola vaccine trial and ancillary care (AC) policy evaluation study with data collection periods in Boende, Democratic Republic of the Congo/AC; trial arm 1 and trial arm 2 indicate the different trajectories of the two arms in the Ebola vaccine trial. Green boxes indicate scheduled visits during the active trial stage, blue boxes indicate a passive stage where no trial activities were planned. The grey crossbars refer to the data collection periods of the below specified studies: (1) Ebola vaccine trial: collection of demographics and baseline characteristics; (2) Geographical study: mapping of participants’ village of residence; (3) AC policy in Ebola vaccine trial: informed consent procedure and policy evaluation study enrolment; (4) AC policy evaluation study: Adverse Event collection; (5) AC policy evaluation study: serious adverse event collection; (6) AC policy evaluation study: collection of survey questions during follow-up and safety phone calls in the Ebola vaccine trial; and (7) AC policy evaluation study: collection of direct expenditures of policy implementation costs.

This was a longitudinal cohort study, nested in an open-label, single-centre, randomised Ebola vaccine trial (ClinicalTrials.gov: NCT04186000; figure 1) conducted among healthcare providers (HCP). In the context of this study, HCPs were categorised into two groups. The first group comprised facility-based healthcare personnel who may be exposed to infectious diseases through their professional activities. It included doctors, nurses, midwives, lab technicians, health facility cleaners and others. The second consisted of community-based healthcare personnel; professionals who may be exposed in community settings and included community healthcare workers, first aid workers, stretcher bearers and similar roles. The main trial was conducted in the General Reference Hospital (GRH) of Boende (hereafter referred to as the trial site), in the Tshuapa Province of the DRC.^2^

### Consenting procedure

The AC policy was introduced in the Ebola vaccine trial in November 2021, before the start of year 3 (figure 1). All trial participants still enrolled at that time point were informed of the policy and enrolled for the policy evaluation study (figure 1, crossbar 3). Consenting to the AC policy was not mandatory to remain in the trial. Nevertheless, all participants consented and potentially benefited from the policy until the trial’s conclusion. No formal sample size calculation was performed for the policy evaluation study, which included all consenting participants.

### The AC policy in practice

The AC policy outcomes were as follows: (1) provision of medication and/or certain diagnostic tests (eg, malaria rapid diagnostic test) from the study pharmacy; (2) direct payment of medical invoices (eg, for consultations or surgical interventions at GRH Boende or elsewhere) or of medication obtained from another pharmacy (eg, if the required medication was unavailable in the study pharmacy); (3) reimbursement of medical invoices (eg, when the participant prefinanced medication, consultations, surgical interventions or hospitalisation costs) and (4) no support possible (eg, no proof of payment). Only healthcare services provided by formal health structures were covered by the AC policy, as the researchers had insufficient legitimacy and knowledge regarding local traditional medicine practices.^9^

Before AC policy implementation, (S)AE were managed on a case-by-case basis. On policy implementation, all newly reported (S)AE were systematically approached as per the AC policy and related algorithm.^3^ As such, SAE that had occurred before policy implementation could be supported as well, on availability of proof of payment. Once an (S)AE was reported during a scheduled (transport reimbursed) or unscheduled (transport not reimbursed) trial visit, the AC algorithm would determine if the medical event qualified for support.

### Patient and public involvement

This evaluation study was set up to quantitatively assess the implementation, use and feasibility of the AC policy in the Ebola vaccine trial in Boende, DRC. The protocol of the evaluation study also included a qualitative component, that is, participant and staff acceptability, through focus group discussions, surveys and interviews. However, the latter aspects will be published later. The study results will be presented to the national EC in the DRC in April 2024.

### Data collection

Data from different sources were combined. First, participants’ demographic information was obtained from the Ebola vaccine trial database (figure 1, crossbar 1). Second, the coordinates of participants’ residence village were collected as part of an ecologic substudy and obtained from its database (figure 1, crossbar 2). Third, information on medical and financial support provided through the AC policy was collected. For AE, this was done prospectively (after AC policy implementation). For SAE, this was done both retrospectively (before AC policy implementation) and prospectively (after implementation) (figure 1, crossbars 4 and 5). The different AC approach for SAE and AE was based on their distinctive characteristics. Irrespective of a causality link to the IP, participants were advised to report all AE, related or unrelated to the IP, as per the AC policy and required to report all SAE, related or unrelated to the IP as soon as possible, as per the main trial procedures. (S)AE definitions used within the trial and AC policy study (online supplemental material 2) were according to the International Conference on Harmonisation (ICH) E2A clinical safety data management scientific guideline.^10^ Each individual (S)AE could have been treated at multiple healthcare facilities, using different treatment methods or at different time points, often leading to multiple support outcomes. Fourth, 6 months after booster vaccination, trial arm 2 participants were invited to partake in a short telephone survey, with multiple choice and open-ended questions that enquired (a) whether AC support was requested and received for experienced medical events, (b) if not, the reason(s) why and (c) any change in residence (to update the geographical database) (figure 1, crossbars 2 and 6). Participants of Arm 1 were not contacted as this telephone visit was not in their trial schedule. Finally, all direct expenses related to the AC policy were aggregated throughout the trial as a part of the financial project administration (figure 1, crossbar 7).

10.1136/bmjgh-2024-015259.supp2 Supplementary data

### Data analysis

Demographic and baseline characteristics and (S)AE data (ie, number of (un)related (S)AE, place of treatment, support provided via the AC policy, reasons for not using the policy) were tabulated and summarised with descriptive statistics (n (%), mean (SD) or median (range)). A binary logistic regression was performed to assess whether age, sex, profession, medical history or the randomisation arm influenced participants’ reporting of AE for AC policy support (yes or no). Professions were categorised as facility-based or community-based HCP.

Incidence rates were calculated for all reported (S)AE. Negative binomial regression models, assessing the mean number of AE reported per day during the trial stage (active or passive) and visit type (scheduled or unscheduled), were used to assess when AE were more frequently reported for AC support. Active trial stages denote a time frame during which trial visits were scheduled (full trial staff capacity on site) while passive stages represent periods with no planned trial activities (less staff present).

To identify the distance between the participants’ residence village and the trial site, the residence villages were geographically mapped with the trial site at the centre of circles that had a 1 km, 5 km, 10 km, 20 km, 30 km and 40 km radius. Unidentified villages were considered missing data. Within Boende, the capital city of the Tshuapa province, a more detailed distinction was made to include the different communes (eg, Motema Mosantu, Marie Louise).

The telephone survey assessed whether participants felt they lived too far from the trial site to report AE for AC support. This was then compared with the actual residence distance, using Fisher’s exact tests. To compare the proportion of participants using the AC policy during scheduled and unscheduled visits per residence location (≤1 km, >1 to 5 km, >10 to 20 km, >20 to 30 km, >30 to 40 km and >40 km), a two-sample z-test for proportions was used. It was impossible to assess the difference against a >5 to 10 km radius as no participants living within this area reported unsupported AE.

All data analyses were performed by using R software V.4.3.1. For the geographical mapping, the package OpenStreetMap V.0.3.4 was used, with osm-public-transport, and circles indicating the distance from the trial site were added using the packages rgeos V.0.6–4 and sp V.2.0–0.

Finally, a post-trial cost analysis focusing on the management of (S)AE was conducted. Data were obtained from the trial’s accounting records and included all direct expenditures related to (S)AE management, that is, medication and diagnostic tests from the study pharmacy, direct payment of medical invoices for medical interventions at the trial site or for medication from external pharmacies, and reimbursement of medical invoices from other health facilities prefinanced by participants.

## Results

### (S)AE reporting

In total, 655 participants were enrolled in the policy evaluation study. Overall, 393 participants (60.0%) reported at least one (S)AE. Multiple (S)AE could be reported at one time point. For AE, 370 participants (56.5%) reported a total of 703 cases (2.4% related, either to the IP (n=16) or to trial participation (n=1)). Overall, 196 participants reported (an) AE once (53.0%), 97 twice (26.2%) and 37 thrice (10.0%), whereas 40 participants used the policy 4–10 times (10.8%). For SAE, 61 participants (9.3%) collectively experienced 94 SAE from enrolment in the trial (18 December 2019) until the conclusion of the trial (12 October 2022). One SAE (postbooster vaccination fever case, identified as ≥40.0°C) was considered related to the IP and two were considered related to trial participation (ie, motorcycle accidents while travelling to or from the site for a scheduled trial visit).^9 11^

The trial stage (active or passive follow-up) influenced the mean daily number of AE reporting (p<0.001; online supplemental table 1A). Overall, 467 AE (66.4%) were reported during the active trial follow-up stage (n=321 participants) and 236 (33.6%) during the passive trial stage (n=142 participants). The daily reporting rate of AE was reduced by 78.8% (95% CI 71.3% to 84.3%) during the passive versus the active trial stage.

10.1136/bmjgh-2024-015259.supp1 Supplementary data

Likewise, the type of trial visit (scheduled or unscheduled) influenced the mean daily number of AE reporting (p<0.001; online supplemental table 1B). For a total of 370 participants reporting AE for AC support, 164 reported them during scheduled visits and 274 during unscheduled visits, with 68 of them reporting AE during both scheduled and unscheduled visits. The daily reporting rate of AE was reduced by 97.4% (95% CI 90.7% to 99.7%) during unscheduled vs scheduled visits.

### Demographic and baseline characteristics

Of the 655 enrolled participants, 395 participants were community-based HCP (60.3%) and 260 were facility-based HCP (39.7%). The odds of reporting AE were not influenced by age (OR 1.01; 95% CI 1.00 to 1.02), nor by medical history at enrolment (OR 1.01; 95% CI 0.66 to 1.53). Compared with women and community-based HCP, men and facility-based HCP had 35.2% (95% CI 4.0% to 56.6%) and 32.3% (95% CI 5.8% to 51.4%) less likelihood of reporting AE for AC support, respectively. The odds of reporting AE for AC support were 2.12 (95% CI 1.54 to 2.92) times higher for arm 2 participants compared with arm 1 participants. However, arm 2 participants had twice the amount of scheduled visits compared with arm 1 participants (two scheduled visits vs one scheduled visit). Consequently, 148 AE were reported in arm 2 during scheduled visits compared with 41 AE in arm 1. For unscheduled visits, arm 1 and arm 2 participants reported a similar number of AE (48.8% (n=251) vs 51.2% (n=263), respectively) (table 1, online supplemental tables 2 and 3).

**Table 1:** Demographics and baseline characteristics of participants in the ancillary care (AC) policy evaluation study, Boende, Democratic Republic of the Congo

	Participants enrolled in AC policy evaluation study (N=655)	Participants with reported AE (N=370)	Participants without reported AE (N=285)	Participants with SAE (N=61)	Survey participants with reported unsupported AE (N=111)
**Race, n (%)**					
Black	655 (100.0)	370 (100.0)	285 (100.0)	61 (100.0)	111 (100.0)
**Sex, n (%)**					
Male	508 (77.6)	280 (75.7)	228 (80.0)	44 (72.1)	90 (81.1)
Female	147 (22.4)	90 (24.3)	57 (20.0)	17 (27.9)	21 (18.9)
**Age**					
Median (range)	46.0 (19.0–75.0)	47.0 (19.0–74.0)	45.0 (20.0–75.0)	45.0 (21.0–68.0)	47.0 (20.0–65.0)
Mean (SD)	45.2 (11.9)	45.5 (12.1)	44.6 (11.6)	44.7 (11.5)	46.1 (10.9)
Profession all categories, n (%)*					
Community health worker*	225 (34.4)	122 (33.0)	103 (36.1)	13 (21.3)	37 (33.3)
Nurse†	170 (26.0)	83 (22.4)	87 (30.5)	19 (31.2)	36 (32.4)
First aid worker*	161 (24.6)	109 (29.5)	52 (18.3)	18 (29.5)	22 (19.8)
Hygienist†	36 (5.5)	23 (6.2)	13 (4.6)	4 (6.6)	3 (2.7)
Midwife†	28 (4.3)	18 (4.9)	10 (3.5)	2 (3.3)	4 (3.6)
Doctor†	11 (1.7)	5 (1.4)	6 (2.1)	1 (1.6)	1 (0.9)
Health facility cleaner†	10 (1.5)	4 (1.1)	6 (2.1)	2 (3.3)	3 (2.7)
Caregiver*	7 (1.1)	2 (0.5)	5 (1.8)	1 (1.6)	3 (2.7)
Lab Technician†	2 (0.3)	0 (0.0)	2 (0.7)	1 (1.6)	0 (0.0)
Pharmacist aid*	2 (0.3)	2 (0.5)	0 (0.0)	0 (0.0)	1 (0.9)
Other†	3 (0.5)	2 (0.5)	1 (0.4)	0 (0.0)	1 (0.9)
**Medical history, n (%)**					
Yes	125 (19.1)	73 (19.7)	52 (18.3)	12 (19.7)	96 (86.5)
No	530 (80.9)	297 (80.3)	233 (81.8)	49 (82.3)	15 (13.5)
Unknown	0 (0.0)	0 (0.0)	0 (0.0)	0 (0.0)	0 (0.0)
**Trial arm, n (%)**					
Arm 1	332 (50.7)	156 (41.6)	169 (59.3)	27 (44.3)	0 (0.0)
Arm 2	323 (49.3)	216 (58.4)	116 (40.7)	34 (55.7)	111 (100.0)

n (%), the number (percentage) of the participants corresponding to the demographic or baseline characteristic category.

*Professions categorised as community-based healthcare providers during logistic regression analysis.

†Professions categorised as facility-based healthcare providers during logistic regression analysis.

AE, adverse event; SAE, serious AE; SD, Standard Deviation.

### AC policy support outcomes

For AE, 621 cases (75.3%) were supported with medication from the study pharmacy. For 161 AE (19.5%), medication was either not available in the study pharmacy but obtained from external pharmacies, and/or another form of medical care was provided (eg, consultation at the GRH) and directly paid by the study funds. For 24 AE (2.9%), participants presented their medical invoices and were reimbursed. Two AE (0.2%) were supported with methods initially not foreseen in the policy. First, one participant received a consult when a neuropsychiatrist from UNIKIN was present in Boende. Second, a COVID-19 test was performed by study staff for a participant experiencing symptoms. Finally, for 17 AE (2.1%) treated elsewhere and reported afterwards, reimbursement was not possible because of the unavailability of invoices and/or other proof of payments. However, only one AE did not receive any support. 16 others still partially benefited from the policy through another form of support (eg, study pharmacy medication; online supplemental table 4). During the active trial stage, a lower proportion of AE (69.2%, n=400) was treated with medication from the study pharmacy compared with the passive stage (89.5%, n=221) (p<0.001). Additionally, medication for AE treatment was more frequently sought in external pharmacies, or another form of medical care was provided and paid for directly with study funds (24.9%, n=144) compared with the passive trial stage (6.9%, n=17) (p<0.001). Lastly, no differences were observed for ‘reimbursements of medical invoices’ (p=0.89), ‘other support’ (p=0.88) or for ‘no support possible’ (p=0.06) for AE between both trial stages (table 2).

**Table 2:** Ancillary care (AC) support provided for SAE before/after AC policy implementation (per trial stage) and treatment location/method, Boende, Democratic Republic of the Congo

	Before AC implementation	After AC policy implementation						Overall (before and after)	AE active versus passive trial period	SAE before versus after AC implementation
		Active trial stage		Passive trial stage		Overall after AC policy implement-tation				
**AC support provided, n (%)**	**SAE** **N=44**	**AE** **N=578**	**SAE** **N=24**	**AE** **N=247**	**SAE** **N=11**	**AE** **N=825**	**SAE** **N=35**	**SAE** **N=79**	P value*	P value*
Study pharmacy medication	8 (18.2)	400 (69.2)	6 (25.0)	221 (89.5)	3 (27.3)	621 (75.3)	9 (25.7)	17 (21.5)	**<0.001**	0.59
Direct payment of medical invoices	1 (2.3)	144 (24.9)	6 (25.0)	17 (6.9)	6 (54.6)	161 (19.5)	12 (34.3)	13 (16.5)	**<0.001**	**<0.001**
Reimbursement of medical invoices	15 (34.1)	16 (2.8)	6 (25.0)	8 (3.2)	2 (18.2)	24 (2.9)	8 (22.9)	23 (29.1)	0.89	0.40
No support possible	19 (43.2)	16 (2.8)	5 (20.8)	1 (0.4)	0 (0.0)	17 (2.1)	5 (14.3)	24 (30.4)	0.06	**0.01**
Other	1 (2.3)	2 (0.4)	1 (4.2)	0 (0.0)	0 (0.0)	2 (0.2)	1 (2.9)	2 (2.5)	0.88	1.00
**Treatment location/method, n (%)**	**SAE** **N=40**	**AE** **N=492**	**SAE** **N=20**	**AE** **N=235**	**SAE** **N=12**	**AE** **N=727**	**SAE** **N=32**	**SAE** **N=72**	P value*	P value*
Medical doctor of GRH Boende or trial site	17 (42.5)	453 (92.1)	11 (55.0)	227 (96.6)	9 (75.0)	680 (93.5)	20 (62.5)	37 (51.4)	**0.03**	0.15
Medical doctor or healthcare personnel outside of GRH Boende	18 (45.0)	9 (1.8)	8 (40.0)	6 (2.6)	2 (16.7)	15 (2.1)	10 (31.3)	28 (38.9)	0.72	0.34
Self-medication	1 (2.5)	26 (5.3)	0 (0.0)	2 (0.9)	0 (0.0)	28 (3.9)	0 (0)	1 (1.4)	**0.007**	1.00
Traditional healer/medicine†	3 (7.5)	2 (0.4)	1 (5.0)	0 (0.0)	1 (8.3)	2 (0.3)	2 (6.3)	5 (6.9)	0.83	1.00
Other/unknown	1 (2.5)	2 (0.4)	0 (0.0)	0 (0.0)	0 (0.0)	2 (0.3)	0 (0)	1 (1.4)	0.83	1.00

n (%), the number (percentage) corresponding to a specific subcategory. For each (S)AE, there were multiple treatment locations/methods and AC support outcomes possible.

The AC support provided refers to the type of the support outcome applied at a given time point per reported (S)AE.

The treatment location/method indicates where the reported (S)AE was/were treated.

*Two-sample z-test for proportions or Fisher’s exact test was used where applicable.

†Traditional healer/medicine indicates that a traditional healer was consulted, or that the participant reported to have taken traditional medicine through self-medication.

AE, adverse event; GRH, General Reference Hospital; SAE, serious adverse event.

Overall, 23 SAE cases (29.1%) were supported via the reimbursement of medical expenses bore elsewhere, 17 (21.5%) via study pharmacy medication and 13 (16.5%) via the direct payment of medical expenses (ie, medications or interventions). Two SAE (2.5%) related to trial participation received partial financial support for treatment via traditional medicine practices, as these were preferred by the participants over conventional medicine.^9^ This was provided ad hoc and not foreseen in the AC policy. Unfortunately, 24 SAE (30.4%) could not be (fully) supported because of the unavailability of invoices and/or proof of payments (online supplemental table 4). Most of these cases (n=19) occurred before the AC policy implementation. When comparing the AC support for SAE, the proportion of participants treated with study pharmacy medication before (18.2%, n=8) and after (25.7%, n=17) policy implementation was not significantly different (p=0.59). However, there was an increase in the proportion of medical invoices for SAE that were directly paid by the study funds after policy implementation (34.3%, n=12), compared with before policy implementation (2.3%, n=1; p<0.001), making this the most applied AC support outcome for SAE after policy implementation. Additionally, a significant decrease (43.2%–14.3%, n=19 and n=5, respectively) in the proportion of SAE that could not be (fully) supported was seen after policy implementation (p=0.01). No differences were observed for the ‘reimbursements of medical invoices’ (p=0.40) or ‘other support’ (p=1.00) outcomes before vs after policy implementation.

### Treatment location/method

Despite the long distances that some participants had to travel to reach the study site (online supplemental figure 1) with limited transportation means (eg, dugout canoe, bicycle, motorbike, on foot), a higher proportion of AE was treated at the trial site during the passive trial stage (96.6%, n=227) than during the active stage (92.1%, n=453; p=0.03; table 2). Additionally, more participants reported self-medication to treat AE during the active trial stage (n=26, 5.3%), compared with the passive stage (n=2, 0.9%). No other differences in healthcare-seeking behaviour were observed between the active and passive trial stages for AE (table 2).

For SAE, participants’ healthcare-seeking behaviour in terms of treatment location and methods was similar before and after AC policy implementation.

### Telephone survey outcomes and geographical mapping

**Figure 2:**
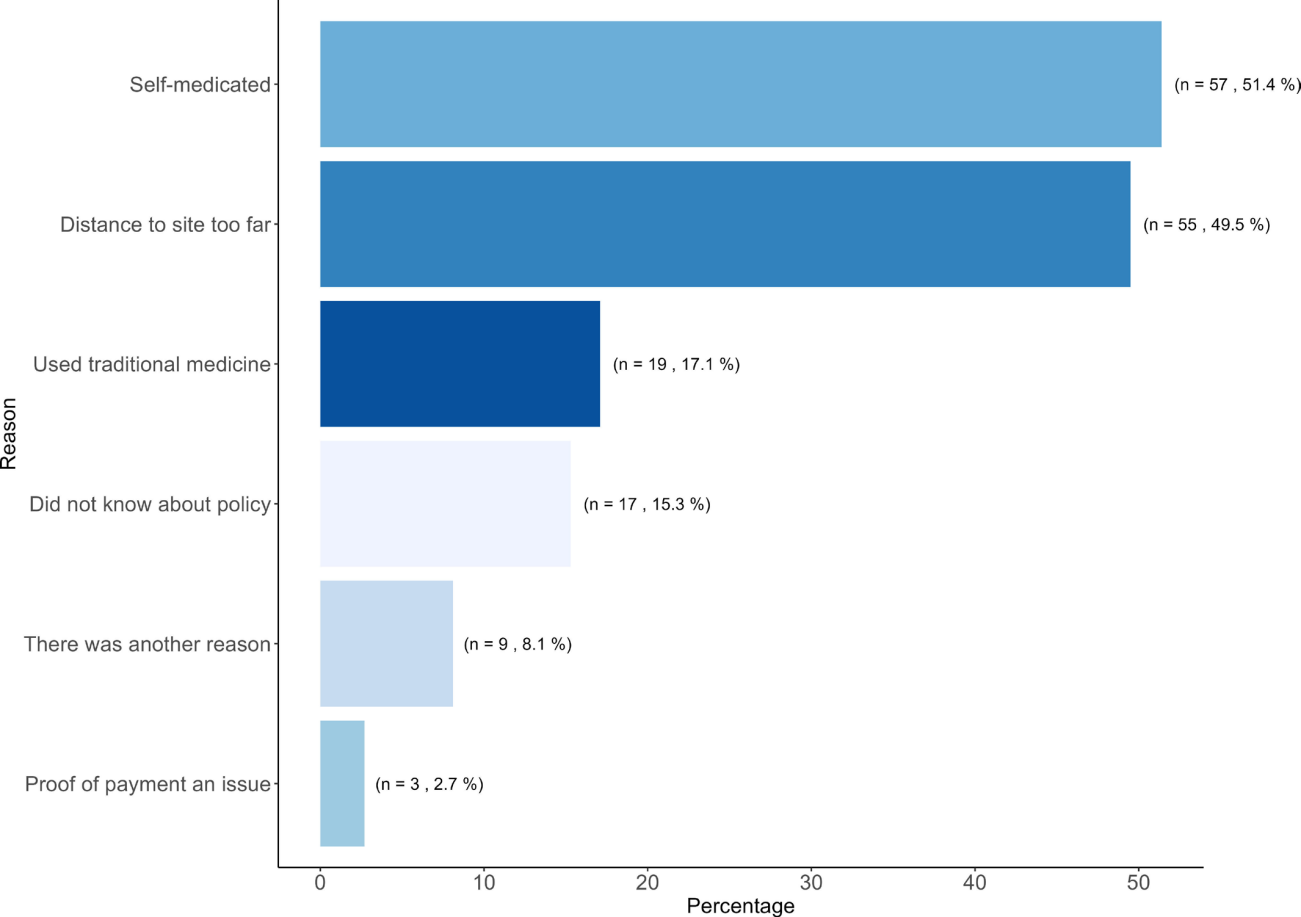
Participants’ reasons for not using/receiving ancillary care policy support for an experienced adverse event (N=111); ancillary care policy evaluation study, Boende, Democratic Republic of the Congo.

A total of 314 participants from arm 2 were contacted by telephone 6 months after booster vaccination. Of them, 311 participated in the AC policy evaluation survey. Of these 311, 111 (35.7%) reported an AE for which support was not sought or was not possible according to the AC policy. Figure 2 shows participants’ reasons for not using/receiving AC policy support when an AE occurred. In total, 57 participants indicated to have self-medicated (51.4%), followed by 55 who perceived the distance to the trial site to be too far (49.6%), and 19 used traditional medicine instead of conventional healthcare covered by the AC policy (17.1%). Although all participants were informed of the AC policy and consented to use it, 17 participants (15.3%) indicated not to know that their AE could have been supported. However, only 3 participants expressed this as the sole reason; 14 did so in combination with other reasons (ie, ‘I live too far’, ‘I had no proof of payment’, ‘I self-medicated’, ‘I used traditional medicine’; online supplemental table 5). Nine participants (8.1%) specified other reasons for not using the AC support, including alternative support provided by the community or other sources,^12^ stock-outs at the study pharmacy, time constraints to travel to the site and long waiting times for consultations at the trial site. Finally, three participants (2.7%) could not be supported because they could not present a proof of payment.

**Figure 3:**
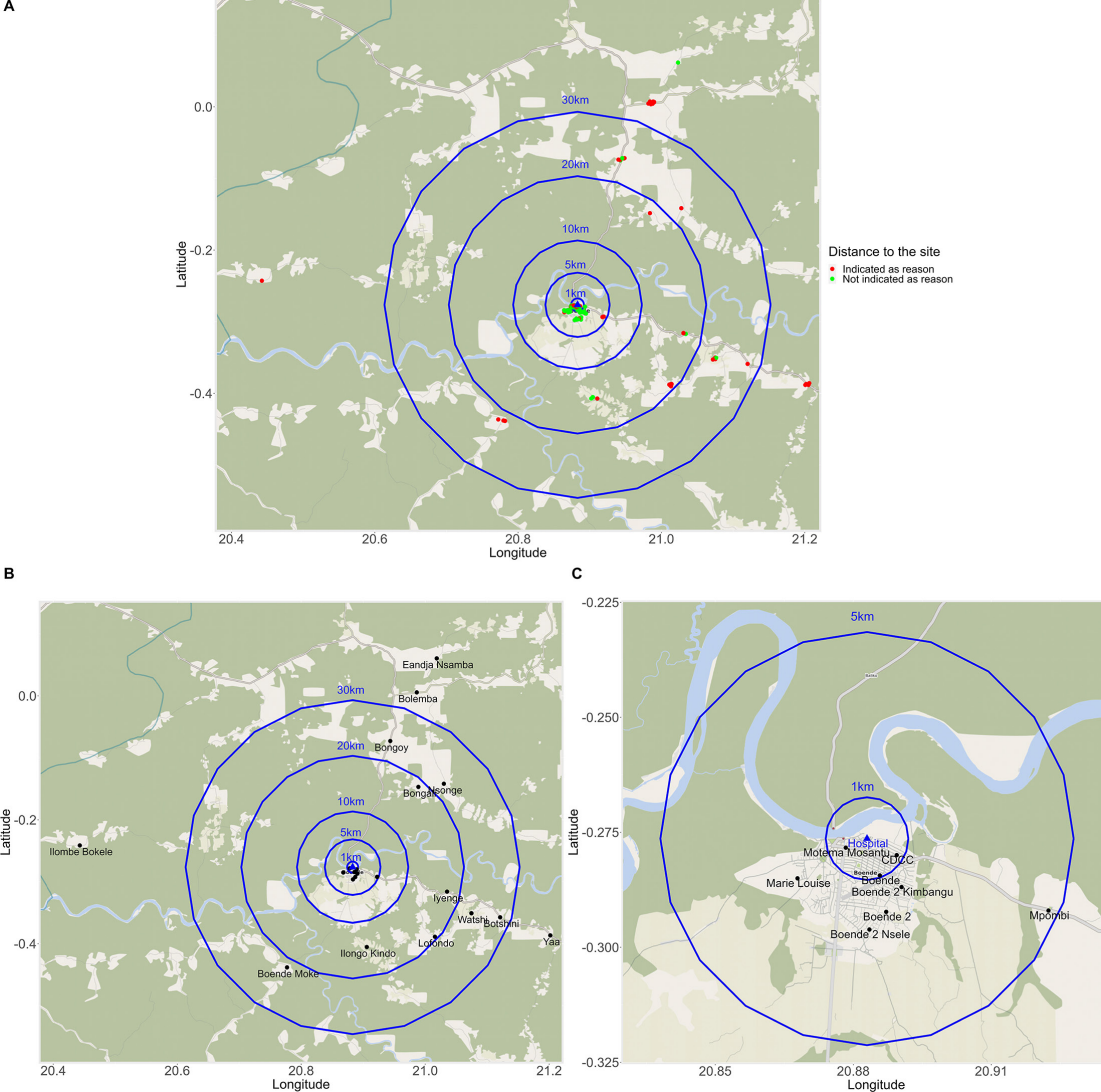
Villages of participants reporting an adverse event for which ancillary care (AC) policy support was not sought or provided (N=111; Boende region, Democratic Republic of the Congo).▲Trial site location; Panel (A) shows the residence location of participants. Red dots represent locations of those who indicated that their residence was “too far” from the study site and therefore did not use the AC policy support for an experienced adverse events (AE). Green dots represent the participants who did not report distance as a reason for non-policy use. The bottom left panel (B), shows the residence village names of participants shown in panel A. In panels (A) and (B), the distance from the site is indicated up to a 30km radius. The bottom righ pannel (C) zooms in at a 5km radius from the site location to show the village names/Boende communes that are not readable on panel (B). Five participants were not included in these plots as they had relocated to the health zones of Bokungu and Mbandaka, located more than 100km distance in radius from the trial site. All but one of these participants indicated distance to the site as the reason for not using the AC policy.

When assessing whether the actual residence distance from the trial site influenced the use of the AC policy, analysis shows that participants living less than 1 km or >1 to 5 km from the site seldomly (n=7 and n=5, respectively) indicated the distance as an issue (figure 3, online supplemental table 6). When comparing the type of visit, participants more frequently used the AC policy during unscheduled (30.7%) vs scheduled visits (16.5%) when living within a 1 km radius from the trial site (p=0.001; online supplemental table 7). However, a higher proportion of AE was reported during scheduled visits compared with unscheduled visits for participants living between >10 and 20 km (24.4% vs 14.6%, p=0.02 and >20 to 30 km (14.6% vs 7.3%, p=0.02) from the site. This was not significant for participants living at a distance of >30 to 40 km and >40 km, but this could be due to the smaller sample size in these groups. Participants living at a >1–5 km distance made use of the policy during scheduled and unscheduled visits similarly (p=0.36). When living more than 10 km from the trial site, between 71.4% and 100.0% of the surveyed participants perceived the distance to the site as an issue (online supplemental table 6). However, 90 participants (32.9%) did travel more than 10 km for AC support during unscheduled visits (online supplemental table 7).

### Cost assessment

On policy implementation, an initial budget of €10 000 from the PI funds was allocated for AC support. The total cost ultimately amounted to €33 196 representing 1.1% of the operational budget available to the PI. When also taking into account the sponsor budget, this percentage dropped to 0.4% of the total study budget, highlighting the limited financial impact of the AC policy for this trial.

The total expenditure included (1) the setup and replenishment of the study pharmacy, created at the start of the trial, independently of the AC policy and (2) the direct payment or reimbursement of AE treatment costs during the AC implementation period, and of SAE during the entire trial duration. In practice, this came down to healthcare sought in the surrounding areas of the participants’ residence location, which is the ‘best care locally available’. The study pharmacy medication took up the largest part of the AC budget (€28 448 or 85.7%), whereas the reimbursement and/or direct payments of medical invoices for both AE and SAE (eg, prefinanced consultations, medication, surgical interventions or hospitalisation) amounted to €4748 (14.3%). However, 41 (S)AE could not be (fully) reimbursed when proof of payment was not available (online supplemental table 4).

## Discussion

We previously described the development process of this study-specific AC policy, as well as some particular implementation challenges which triggered ethical dilemmas regarding insurance coverage gaps for mobility-related injuries, the use of traditional medicine practices and the strong family involvement in participants’ SAE management.^3 9^ Previous studies have assessed stakeholder representatives’ perceptions and experiences of AC or discussed models or decision-making processes involving AC practices.^13–16^ Researchers have called for a quantitative assessment of AC needs and for the evaluation of AC delivery services.^17 18^ Other scholars presented practical case studies or reported on their experiences with the provision of AC when participants developed a life-threatening illness during research, suggesting that AC provision is feasible, if supported by the commitment of investigators and funders.^18 19^ To the best of our knowledge, this is the first formal quantitative evaluation of a study-specific AC approach.

The policy evaluation lasted 11 months. During this period, over half (n=370, 60.0%) of the enrolled trial participants made use of the AC policy. All three initially planned AC support options (ie, study pharmacy medication, reimbursement of medical invoices and direct payment of medical invoices) were used in both AE and SAE management. For AE, the most frequently used support was the provision of medication via the study pharmacy (n=621, 75.3%). For SAE, many costs were directly paid for (n=12, 34.3%) once the policy was implemented.

Providing AC could offer a significant benefit, not only from an ethical perspective, but also from a scientific prospective, as it is likely to result in a more comprehensive reporting of AE. Still, we observed a lower likelihood of AE reporting for facility-based HCP compared with community-based HCP, and for men compared with women. This observation may be due to a difference in educational background and/or the place of work (ie, HCP work in health facilities with access to healthcare services).^20 21^ Additionally, a comprehensive study conducted across 59 countries revealed that women generally use healthcare services at rates equal to or surpassing those of men.^22^ The authors argue that the gender gap in self-reported health appears to be shaped by a combination of societal (eg, disparities in employment and education, coupled with gender inequality) and biological factors.^22^

In this study, arm 2 participants were twice as likely to report AE compared with arm 1 participants. However, as their AE reporting was similar during unscheduled visits, this can largely be explained by the higher number of scheduled visits in year 3 for arm 2 participants compared with arm 1 participants (two vs one scheduled visit, respectively). Additionally, one of the two scheduled visits was 7 days after booster vaccination, which could lead to a higher use of the AC policy if an AE after vaccination was still persisting. Conversely, we estimate that the administration of the vaccine did not greatly influence the AC policy use, as only 2.3% (n=16) of AE and 1.1% (n=1) of SAE were considered related to vaccination. Thus, the population and design of the Ebola vaccine trial impacted the use of the AC policy by the participants. Study findings indicated a 78.8% (95% CI 71.3% to 84.3%) reduction in the daily AE reporting rate during the passive trial stage compared with the active stage and a 97.4% (95% CI 90.7% to 99.7%) reduction during unscheduled versus scheduled visits. Consequently, a trial with more scheduled visits or lengthier active stages is likely to see a higher frequency of participants using the AC policy. However, it is noteworthy that many AE were also reported during unscheduled trial visits (n=514) and the passive follow-up stage of the trial (n=236).

The trial design influenced the application of the AC policy by study personnel. Unexpectedly, study doctors made less use of the study pharmacy medication for AE treatment in the active compared with the passive study stage. Several reasons could apply. First, as reported by participants during the telephone surveys, the study pharmacy experienced periodic stock ruptures which impacted medication availability. Second, more study staff (eg, (co-)PI, financial administrator) was present during the active trial stage, facilitating the obtention and direct payment of medication from external pharmacies (eg, if the preferred medication was unavailable at the study pharmacy) and/or other methods of medical care (eg, consultation at the GRH of Boende) with study funds. Following this, we hypothesise that stock-outs in the study pharmacy during the active study stage were mediated by obtaining medication from external pharmacies or directly paying for other medical care, that is, decreasing the study pharmacy use while alternative medication available in the study pharmacy was provided during stock-outs in the passive study stage, that is, increasing its use.

When it comes to participants’ self-reported reasons for the non-use of AC policy support for AE, just over half of the surveyed participants (n=57, 51.4%) reported to have resolved their AE through self-medication. Considering that the study population were HCP and frontliners, this outcome was not unexpected.^20 23^ It is uncertain, whether other population groups would have self-medicated similarly for AE treatment.

Nearly half of the surveyed participants (n=55, 49.6%) indicated the distance to the study site as a reason for not using the AC policy. On further geographical analysis, we found that distance was more commonly reported as an issue when participants lived >10 km from the study site. Though this may seem relatively near, the local setting needs to be considered: most participants travelled on foot, by bicycle, motorbike or dugout canoe, facing natural obstacles in the environment to reach the study site.^24^ Unfortunately, details on the type of AE (eg, severity or urgency) could not be taken into account in the analysis as they were not collected as part of the survey. A further limitation was that the specific location of the health centres in the area, and their type and quality of healthcare were unknown to the researchers. These aspects could, therefore, not be taken into account during analysis. However, while health centres are typically available at a 5 km travelling distance for DRC’s residents,^24^ we observed that several participants who lived more than 10 km from the site still reported AE for AC support during unscheduled visits (n=90).

Even though the AC policy was thoroughly explained during an informed consent procedure, and all participants consented to it, 17 surveyed participants (15.3%) expressed a lack of awareness regarding the potential support for their AE. Although recall bias could be at play, we suggest, based on the replies to the telephone survey, that interpretation bias may have occurred. To the specific multiple choice question, ‘Why did you not come to the trial for treatment or a reimbursement of expenses?’, 14 out of 17 participants responded ‘I didn’t know it was possible’, in combination with other reasons (ie, ‘I live too far’, ‘I had no proof of payment’, ‘I self-medicated’, ‘I used traditional medicine’). As the latter reasons could indeed not be supported, it is unclear whether participants thought support was not possible because of the other reason, or that they, in general, did not know/recall the AC policy at all. Moreover, language barriers or illiteracy may have contributed as well. This would suggest that consent documents and procedures were either too lengthy and complex, or too hurried, leading to poor understanding and recall.^25–27^

Three elements were initially not integrated in the AC policy, but emerged as needs and were addressed during the trial. First, as the COVID-19 pandemic could not be anticipated, COVID-testing performed by a study physician was not foreseen, but later implemented free of charge when participants presented with symptoms. If tested positive, they were referred to the GRH of Boende for a free consultation, as per local guidelines at the time. Second, conventional psychological and mental healthcare services are not available in Boende. However, a consultation was provided when a neuropsychiatry professor from UNIKIN was in Boende for a trial-related workshop.^28^ Third, some participants turned to traditional medicine for the treatment of their SAE, which were discussed elsewhere.^9^ Altogether, these three experiences point to the necessity of flexible AC procedures, policies and guidelines, that are adaptable to a complex environment or unforeseen elements, such as the COVID-19 pandemic.

Seen the relatively small expenditures for AC support (€33 196; 1.1% of the operational budget and 0.4% of the overall study budget), this evaluation study shows that the health benefits for participants greatly outweighed AC policy costs. Therefore, depending on the trial budget, the attributes of the local health system, participant characteristics (eg, chronically ill participants compared with our generally healthy), and participant needs, our AC policy could be adapted accordingly for other clinical research in resource-constrained settings.

This study had some limitations. First, the demographics and baseline characteristics of the participants were collected at enrolment in the main trial, 2 years before the start of the AC policy evaluation study. As such, parameters such as medical history might have changed. Second, there was a reporting bias, that is, participants who for various reasons did not come to the site for AE reporting and AC policy support, were missed. Only the reported events were included in the policy evaluation. However, this limitation was partially addressed when enquiring about any non-reported AE during the telephone survey, including the reasons for non-reporting. Third, the geographical mapping used to represent distance from the trial site made use of the Euclidean distance only and was not triangulated with social determinants of health (eg, education or socioeconomical background) and other travel barriers that affect access to care (eg, seasonal variation, land use, road network, geographical factors).^29 30^ Fourth, the AC policy was only implemented in the last year of the trial, which may have impacted the assessment of costs related to the reimbursement and/or direct payment of medication. Fifth, the AC policy restricted the provision of healthcare services to those available locally and omitted specialised care available outside of the research setting. This limitation also had implications for the policy’s cost assessment. Lastly, no indirect costs (eg, cost of additional manpower to ensure (S)AE management (of unrelated cases)) were included in the cost analysis.

## Conclusions and recommendations

This study indicates that an AC policy can be introduced in a clinical trial without excessively burdening the research team and local health system. We believe, in light of the high uptake, applicability and financial feasibility of the AC policy, that it is feasible and ethically commendable to implement a study-specific AC policy during clinical trials in resource-constrained settings. This evaluation study demonstrates that the characteristics of the trial design, study population, site accessibility, local context and local health system altogether influence the use and applicability of an AC policy. All possible support options of our trial-specific policy (ie, provision of medication from a study pharmacy, and direct payment or reimbursement of medical invoices of locally available healthcare services) were crucial in providing adequate, equal and systematic medical care for (S)AE to trial participants. The policy was most applied for AE with the provision of medication from the study pharmacy. Our findings can inform the development of study-specific AC policies for other clinical trials in resource-constrained settings, in order to reconcile the achievements of research objectives with the protection of the health and well-being of participants. We hope that the results of this study can inspire and motivate policy-makers, national EC and funders to require feasible but adequate AC measures in global health research.

## Data Availability

Data are available on reasonable request.

## Data availability statement

Individual deidentified participants’ data and a data dictionary will be made available to others in the scientific community on request after publication of this study. Standard criteria for making data available for valid research projects will be used, following application by suitably qualified researchers and upon presentation of a defined analysis plan to the corresponding author.
