# Supplementary material for: An ancillary care policy in a vaccine trial conducted in a resource-constrained setting: evaluation and policy recommendations
Source: BMJ Glob Health. 2024 Jun 10;9(6):e015259. doi: 10.1136/bmjgh-2024-015259 (PMC11168174; doi:10.1136/bmjgh-2024-015259)
Supplement: Supplementary data [file bmjgh-2024-015259supp002.pdf]

UNIVERSITE DE KINSHASA
FACULTE DE MEDECINE
DEPARTEMENT DE MEDECINE TROPICALE

Supplementary Material:
Summary of Algorithm Policy for Non-Related (Serious) Adverse Events

An algorithm policy for Non-related (Serious) Adverse Events was developed for implementation in the clinical trial in Boende, DR Congo (ClinicalTrials.gov Identifier: NCT04186000). In what follows, the definitions of the used terminology, inclusion and exclusion criteria and the support provided under the algorithm policy are outlined.

1. Definitions

An *Adverse Event* (AE) refers to any untoward medical occurrence in a participant of a clinical study whom was administered a medicinal (investigational or non-investigational) product.

A *Serious Adverse Event* (SAE) is any untoward medical occurrence which at any dose:

- results in death;
- is life-threatening;
- requires inpatient hospitalisation or prolongation of existing hospitalisation;
- results in persistent or significant disability/incapacity;
- is a congenital anomaly/birth defect;
- is a suspected transmission of any infectious agent via a medicinal product;
- or is medically important.

In general, the diagnosis and/or interpretation by a medical doctor of the above criteria will be essential to determine and assess each medical event separately, with a benevolent approach to its treatment and support.

The medical event is considered to be *non-related* when there is no relationship between the medical condition and the study. It can both refer to the study drug and to participation in the study itself.

In this trial, all AEs are managed equally, regardless of relatedness to the investigational product. In case an SAE is considered to be *related*, be it to the study drug or to study participation, it will be covered by the insurance of the Sponsor. The algorithm policy is directed to treat and financially cover the treatment expenses of non-related AEs and non-related SAEs.

2. Inclusion criteria

To be eligible for support via this algorithm policy, the following criteria apply:

- He/she who has encountered the AE or NR-SAE is an enrolled participant in the abovementioned Ebola vaccination trial.
- The medical condition complies with one of the above mentioned cases of what is considered a(n) (S)AE.
- The (S)AE is not related to the study drug or to study participation.
- When a participant suffers from a (prior) disease or condition, a possible complication of this condition that results in an NR-(S)AE can be supported.

UNIVERSITE DE KINSHASA
FACULTE DE MEDECINE
DEPARTEMENT DE MEDECINE TROPICALE

3. Coverage of the algorithm policy:

- In case of an AE, whether related or unrelated to the study intervention, medication is provided by the study's pharmacy. If necessary additional diagnostic tests are performed. In case the AE was not treated at the trial facilities, reimbursement of the medication costs is possible upon the next scheduled study visit, after presenting proof of payment.
- The financial support (or refund) of direct costs related to a NR-SAE involves:
    - hospitalization fees,
    - costs of the medical interventions needed or taken,
    - doctor fees,
    - and/or the cost of disposables and medical consumables.
- In the event that treatment of the participant is no longer required (e.g. treatment has been done elsewhere than at the study site) or can no longer be given (e.g. the participant has deceased), a refund of the endured costs is possible when the NR-SAE is confirmed and the medical interventions are assessed relevant. Reimbursement or payment shall be done upon presentation of the necessary proof of payments or invoices and to a maximum according to the fixed tariffs for medical costs applicable at the General Reference Hospital of Boende.
- Possible additional arrangements for the participant's transport to the study site, should that be required, so that the participant can continue with the trial activities (e.g. person with leg fracture).

4. What is not covered by the algorithm policy:

- Hospitalisations that were planned before enrolment in the trial, whereby the condition has not worsened. (A prolongation of a planned hospitalisation will be treated as a new SAE.)
- Support of medication that is not on the Study Pharmacy List;
- Possible long-term consequences of a NR-SAE;
- Indirect costs (being other than those related to the immediate treatment of the NR-SAE); e.g. burial costs, loss of wage, etc.

5. Duration of coverage

The algorithm policy and its benefits for enrolled participants lasts 30 months in total, starting on the day of enrollment, which covers the duration of the study activities.

* * *
