# Supplementary material for: An ancillary care policy in a vaccine trial conducted in a resource-constrained setting: evaluation and policy recommendations
Source: BMJ Glob Health. 2024 Jun 10;9(6):e015259. doi: 10.1136/bmjgh-2024-015259 (PMC11168174; doi:10.1136/bmjgh-2024-015259)
Supplement: Supplementary data [file bmjgh-2024-015259supp001.pdf]

Supplementary material – Ancillary Care Policy Evaluation Study

Supplementary material

Tables

Table 1a. Negative binomial regression model assessing the average number of visits per day adjusted for trial stage; ancillary care policy evaluation study, Boende, Democratic Republic of the Congo.

	Estimate	Std. Error	P-value
(Intercept)	1.60	0.12	**<0.001**
Passive stage	-1.55	0.15	**<0.001**

Table 1b. Negative binomial regression model assessing the average number of visits per day adjusted for type of visit; ancillary care policy evaluation study, Boende, Democratic Republic of the Congo.

	Estimate	Std. Error	P-value
(Intercept)	4.14	0.64	**<0.001**
Unscheduled visit	-3.66	0.65	**<0.001**

Table 2. Odds ratios with 95% confidence intervals per predictor as per the logistic regression model (outcome: reporting of adverse events Yes or No); ancillary care policy evaluation study, Boende, Democratic Republic of the Congo.

	OR	2.5%	97.5%
**(Intercept)**	0.98	0.48	2.00
Profession facility-based HCP	0.68	**0.49**	**0.94**
Gender Male	0.65	**0.43**	**0.96**
Age	1.01	1.00	1.02
Medical history Yes	1.01	0.66	1.53
Study Arm 2	2.12	**1.54**	**2.92**

HCP= Healthcare provider; OR = Odds ratio

Table 3. Adverse event reporting rate according to the visit type per trial arm; ancillary care policy evaluation study, Boende, Democratic Republic of the Congo.

Trial arm, n (%) – number of days where this type of visit applied	Scheduled visits (N = 189)	Unscheduled visits (N = 514)	All visits (N = 703)
Arm 1	41 (21.7) – 1 day	251 (48.8) – 318 days	292 (41.5)
Arm 2	148 (78.3) – 2 days	263 (51.2) – 318 days	411 (58.5)

Lemey G, *et al. BMJ Glob Health* 2024; 9:e015259. doi: 10.1136/bmjgh-2024-015259

Supplementary material – Ancillary Care Policy Evaluation Study

Table 4. (Serious) adverse events with 'no support possible' as outcome; ancillary care policy evaluation study, Boende, Democratic Republic of the Congo.

AC (non-)support outcome(s), n (%)	AE (N = 17)	SAE with 'no support possible' outcome, according to timepoint of AC policy implementation		
		Before policy implementation (N=19)	After policy implementation (N=5)	Overall (N=24)
No invoice or proof of payment	1 (5.9)	12 (63.2)	3 (60.0)	15 (62.5)
Combination 1:<br>• No invoice or proof of payment<br>• BUT direct payment of medical expenses	3 (17.6)	0 (0.0)	0 (0.0)	0 (0.0)
Combination 2:<br>• No invoice or proof of payment<br>• BUT medication from study pharmacy	9 (52.9)	6 (31.6)	2 (40.0)	8 (33.3)
Combination 3:<br>• No invoice or proof of payment<br>• BUT direct payment of medical expenses<br>• BUT medication from study pharmacy	4 (23.5)	0 (0.0)	0 (0.0)	0 (0.0)
Combination 4:<br>• No invoice or proof of payment<br>• BUT medication from study pharmacy<br>• BUT reimbursement of medication expenses	0 (0.0)	1 (5.3)	0 (0.0)	1 (4.2)

AC = ancillary care, AE = adverse event; SAE = serious adverse event; N = the total number of events with 'no support possible'; n (%) = the number (percentage) of events corresponding to a specific sub-category of 'no support possible'

Lemey G, *et al. BMJ Glob Health* 2024; 9:e015259. doi: 10.1136/bmjgh-2024-015259

Supplementary material – Ancillary Care Policy Evaluation Study

Table 5. Amount of survey participants indicating 'I did not to know support was possible', in combination with other reasons for non-support; ancillary care policy evaluation study, Boende, Democratic Republic of the Congo.

**Surveyed participants' reasons for non-support of an AE (N=17)**	
**Support outcome**	**n (%)**
I did not know support was possible	3 (17.6)
Combination 1:<br>• I did not know support was possible<br>• AND I live too far	6 (35.3)
Combination 2:<br>• I did not know support was possible<br>• AND I did not have no proof of payment	1 (5.9)
Combination 3:<br>• I did not know support was possible<br>• AND I self-medicated	3 (17.6)
Combination 4:<br>• I did not know support was possible<br>• AND I used traditional medicine	1 (5.9)
Combination 5:<br>• I did not know support was possible<br>• AND I live too far<br>• AND I self-medicated	3 (17.6)

AE = adverse event; N = the total number of participants that indicated having an AE for which no support was possible/sought from the AC policy; n (%) = the number (percentage) of participants corresponding to a specific sub-category.

Lemey G, *et al. BMJ Glob Health* 2024; 9:e015259. doi: 10.1136/bmjgh-2024-015259

Supplementary material – Ancillary Care Policy Evaluation Study

Table 6. Comparison of perception of distance as "too far" to the trial site versus the distance based on residence coordinates; ancillary care policy evaluation study, Boende, Democratic Republic of the Congo.

Distance	≤1km	>1-5km	>10-20km	>20-30km	>30-40km	>40km	N
≤1km	7 (25.0)	0.52	**0.004**	**<0.001**	**<0.001**	**0.03**	28
>1-5km	FE	5 (16.1)	**<0.001**	**<0.001**	**<0.001**	**0.008**	31
>10-20km	FE	FE	11 (73.3)	0.69	0.09	1.00	15
>20-30km	FE	FE	FE	13 (81.3)	0.23	0.62	16
>30-40km	FE	FE	FE	FE	14 (100.0)	0.10	14
>40km	FE	FE	FE	FE	FE	5 (71.4)	7

N = total number of participants that experiences an adverse event for which treatment was not sought or possible per actual residence distance to the site; Diagonally, the n (%) – number (percentage) – of participants indicating the distance as too far to use ancillary care support; below the diagonal the used statistical test is shown; FE = Fisher Exact test; above the diagonal the p-value of test FE test shown.

Table 7. Distance of residence from the study site for participants with adverse events; ancillary care policy evaluation study, Boende, Democratic Republic of the Congo.

	Participants with AE reported to the site (N = 370)	Arm 1 and Arm 2 participants coming for unscheduled visits (N=274)	Arm 1 and Arm 2 participants coming for scheduled visits (N=164)	Unscheduled vs scheduled*	Arm 2 participants not using/supported by AC policy for an AE (N=111)
**Residence distance**	**n (%)**	**n (%)**	**n (%)**	**p-value**	**n (%)**
≤1km	100 (27.1)	84 (30.7)	27 (16.5)	**0.001**	28 (25.2)
>1-5km	109 (29.5)	88 (32.1)	45 (27.4)	0.36	31 (27.9)
>5-10km	5 (1.4)	5 (1.8)	0 (0.0)	-	0 (0.0)
>10-20km	63 (17.0)	40 (14.6)	40 (24.4)	**0.02**	15 (13.5)
>20-30km	35 (9.5)	20 (7.3)	24 (14.6)	**0.02**	16 (14.4)
>30-40km	28 (7.6)	15 (5.5)	16 (9.8)	0.13	14 (12.6)
>40km	20 (5.4)	15 (5.5)	9 (5.5)	1.00	7 (6.3)
Unknown	10 (2.7)	7 (2.6)	3 (1.8)	-	0 (0.0)

*AC= ancillary care; AE = adverse event; N = the total number of participants with an AE reported for which support was sought; n (%) = the number (percentage) of participants corresponding to a specific sub-category; *Two-sample z-test for proportions*

Lemey G, *et al. BMJ Glob Health* 2024; 9:e015259. doi: 10.1136/bmjgh-2024-015259

Supplementary material – Ancillary Care Policy Evaluation Study

Figure

Supplementary Figure 1. Villages of participants indicating to have had an Adverse Event for which Ancillary Care policy support was sought (N=370; Boende region, Democratic Republic of the Congo)

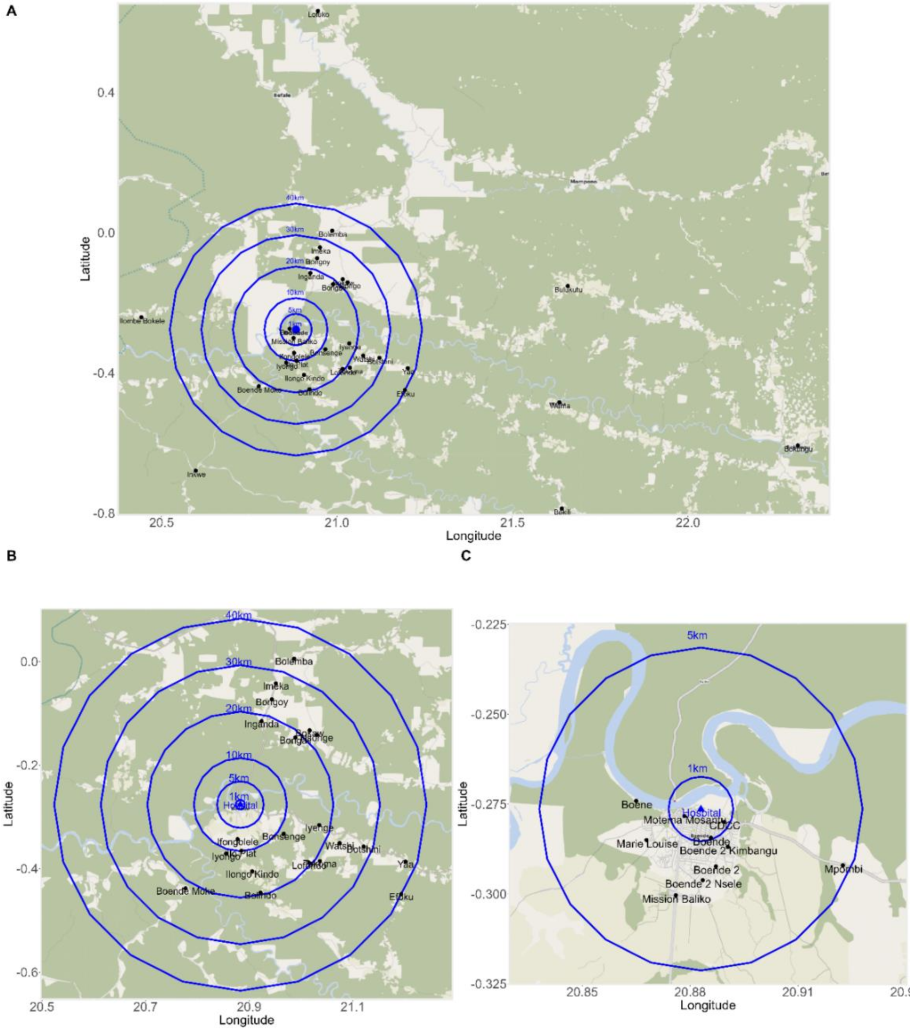

▲ *Trial site location; The upper panel (A) shows all villages in the Boende health zone, or its surrounding health zones (Befale, Wema, and Bokungu), from where participants travelled to obtain medical and/or financial support for (an) AE(s). Participants living further than the surrounding health zones (N=6), or for which the village of residence was unknown (N=3), were not included in these analyses. For six additional villages (five in the Boende health zone, and one in the Wema health zone) coordinates could not be obtained. The lower left panel (B) zooms in at a 40km radius from the site location to show the village names that were not readable on panel A. The lower right panel (C) zooms in at a 5km radius from the site location to show the villages and Boende communes that were not readable on panel B.*

Lemey G, *et al. BMJ Glob Health* 2024; 9:e015259. doi: 10.1136/bmjgh-2024-015259
